# LDER-GE estimates phenotypic variance component of gene–environment interactions in human complex traits accurately with GE interaction summary statistics and full LD information

**DOI:** 10.1093/bib/bbae335

**Published:** 2024-07-09

**Authors:** Zihan Dong, Wei Jiang, Hongyu Li, Andrew T DeWan, Hongyu Zhao

**Affiliations:** Department of Biostatistics, Yale School of Public Health, 60 College Street, New Haven, CT 06510, United States; Center for Perinatal, Pediatric and Environmental Epidemiology, 60 College Street, Yale School of Public Health, New Haven, CT 06510, United States; Department of Biostatistics, Yale School of Public Health, 60 College Street, New Haven, CT 06510, United States; Department of Biostatistics, Yale School of Public Health, 60 College Street, New Haven, CT 06510, United States; Center for Perinatal, Pediatric and Environmental Epidemiology, 60 College Street, Yale School of Public Health, New Haven, CT 06510, United States; Department of Chronic Disease Epidemiology, Yale School of Public Health, 60 College Street, New Haven, CT 06510, United States; Department of Biostatistics, Yale School of Public Health, 60 College Street, New Haven, CT 06510, United States

**Keywords:** gene–environment interaction, genome-wide interaction scan, missing heritability, linkage disequilibrium, statistical efficiency

## Abstract

Gene–environment (GE) interactions are essential in understanding human complex traits. Identifying these interactions is necessary for deciphering the biological basis of such traits. In this study, we review state-of-art methods for estimating the proportion of phenotypic variance explained by genome-wide GE interactions and introduce a novel statistical method Linkage-Disequilibrium Eigenvalue Regression for Gene–Environment interactions (LDER-GE). LDER-GE improves the accuracy of estimating the phenotypic variance component explained by genome-wide GE interactions using large-scale biobank association summary statistics. LDER-GE leverages the complete Linkage Disequilibrium (LD) matrix, as opposed to only the diagonal squared LD matrix utilized by LDSC (Linkage Disequilibrium Score)-based methods. Our extensive simulation studies demonstrate that LDER-GE performs better than LDSC-based approaches by enhancing statistical efficiency by ~23%. This improvement is equivalent to a sample size increase of around 51%. Additionally, LDER-GE effectively controls type-I error rate and produces unbiased results. We conducted an analysis using UK Biobank data, comprising 307 259 unrelated European-Ancestry subjects and 966 766 variants, across 217 environmental covariate-phenotype (E-Y) pairs. LDER-GE identified 34 significant E-Y pairs while LDSC-based method only identified 23 significant E-Y pairs with 22 overlapped with LDER-GE. Furthermore, we employed LDER-GE to estimate the aggregated variance component attributed to multiple GE interactions, leading to an increase in the explained phenotypic variance with GE interactions compared to considering main genetic effects only. Our results suggest the importance of impacts of GE interactions on human complex traits.

## Introduction

A growing body of literature underscores the significant role of gene–environment (GE) interactions in shaping human complex traits [1–4]. The exploration of GE interactions may elucidate a portion of the “missing heritability” [5]—the phenotypic variance not accounted for by known genetic effects. Additionally, the inference of GE interactions and their effects can contribute to our understanding of human disease etiology and mechanisms [6], and enhance our ability to assess risk and identify high-risk individuals, ultimately supporting the development of personalized medicine [1]. Traditionally, environmental exposure variables have been limited to factors like environmental toxins, air pollutants, or viral infections [6]. However, some gene–environment interaction studies [7, 8] also consider other variables, heritable or non-heritable, such as sex, as environmental exposure variables. In this study, we adopt a broad definition, considering both nonheritable covariates and heritable phenotypes as environment interactive variables, as previously discussed [2].

Numerous methods and tools have been developed to investigate GE interactions from various angles. One such approach is the genome-wide interaction scan (GWIS), which estimates the interaction effect [9] between individual genetic variants and environmental factors through regression. GWIS generates interaction summary statistics for each variant, akin to conventional genome-wide association studies (GWASs). However, we note that GE interaction effect sizes tend to be smaller than genetic main effects [10]. Consequently, this can lead to reduced statistical power, particularly when challenged by the multiple testing burden across the entire genome [11]. Zhu *et al*. advanced the Single Nucleotide Polymorphism (SNP)-level GxE analysis [12] by proposing a mendelian-randomization-like approach to leverage results from GWAS and GWIS from potentially different samples to boost the GxE effect detection at the SNP level and showed substantial improvement in real data analysis. Zhu *et al*. successfully detected several GxSmoking and GxDrinking signals on lipids traits, which were missed by traditional GWIS. Several studies have directed their efforts toward estimating the genome-wide contribution of GE interactions through diverse statistical approaches. One such method is the Gene–Environment Interaction Genomic Restricted Maximum Likelihood (GEI-GREML), which leverages restricted maximum likelihood estimation by precomputing the correlation matrix of the GE term across samples [13]. On the other hand, the Multivariate Reaction Norm Model (MRNM) is a reaction norm model that has the capability to distinguish between GE interaction and GE correlation [14]. Both GEI-GREML and MRNM necessitate individual-level genotype data and can be computationally demanding and time-consuming, especially when dealing with extensive biobank datasets.

To tackle these challenges, researchers have devised alternative methods that make use of GWIS summary statistics. Notably, methods like PIGEON [7] and GxEsum [8] build upon the principles of linkage disequilibrium (LD)–score regression (LDSC) [15]. They harness partial LD information among genetic variants to estimate the phenotypic impact of GE interactions using the method of moments. However, this approach often results in reduced statistical efficiency when estimating variance components, because the phenotypic variance attributed to GE interactions is often considerably smaller than the narrow-sense heritability. For example, across an analysis encompassing more than 500 traits, the phenotypic variance explained by genetic–sex interactions typically falls within the range of 0% to a maximum of 2% [7]. While this may appear modest, acknowledging and investigating this component remain important for our understanding of complex traits and disease etiology. An inefficient estimation method may fail to detect the contribution of GE interactions. Consequently, there is a need for a more efficient approach to estimate the phenotypic variance explained by GE interactions while effectively managing computational demands. Current LDSC-based frameworks [7, 8, 15] make use of the squared variant LD matrix but primarily focus on diagonal information. Previous research [16, 17] has convincingly shown that incorporating the complete LD information can substantially enhance the efficiency of estimating narrow-sense heritability under the genetic additive effect model. Building upon this insight, we introduce the Linkage-Disequilibrium Eigenvalue Regression for Gene–environment interactions (LDER-GE) to estimate the genome-level GE interaction variance component more efficiently.

LDER-GE mimics the original LDER framework [16], which harnesses the full potential of LD information through eigen-decomposition of the LD matrix. This process transforms the original GWIS summary statistics and consolidates the association information. Notably, LDER-GE relies on summary statistics and the LD matrix constructed using a reference panel. Consequently, it efficiently manages large-scale Biobank data without imposing substantial computational demand. Extensive simulations provide evidence that both LDER-GE and the LDSC-based method effectively control the type-I error rate and deliver unbiased estimates. However, LDER-GE excels in terms of statistical efficiency compared to the LDSC-based method in all simulation scenarios. In a real-data application involving 217 E-Y pairs from the UK-Biobank [18], the LDSC-based method identified 23 GE interaction signals, whereas LDER-GE identified 34 E-Y pairs (48% increase). For a more precise assessment of the contribution of GE interactions to missing heritability, we estimate the aggregated GE interaction variance involving multiple environmental covariates and the analyzed phenotypes. In this regard, LDER-GE facilitates more accurate estimation. In summary, the missing heritability contributed by the aggregated multicovariate GE interaction variance represents a substantial addition to the narrow-sense heritability.

In this paper, we first discuss the strengths and limitations of various statistical methods to estimate the genome-level contribution of GE interaction effects in human complex traits. Then, we introduce the model and method overview of LDER-GE framework and compare it with existing approaches. Next, we report the simulation and real data analysis performance of LDER-GE with its comparative methods. Finally, we interpret the analysis results of LDER-GE regarding the biological significance.

## Results

### Existing methods

Table 1 provides a comprehensive overview of existing statistical approaches to inferring the phenotypic variance of gene–environment (GE) interactions. These methods can be broadly categorized into those that rely on individual-level genotype data and those that utilize summary statistics from GWIS in conjunction with an LD reference panel.

**Table 1:** Comparisons of existing statistical methods to estimate phenotypic variance explained by GE interactions

Methods	Data input	Overview	Computation efficiency	Estimation accuracy	Privacy issue	Other features
GCTA (GCI-GREML [12])	Individual-level genotype	Restricted Maximum Likelihood (REML) estimation in a linear mixed model (LMM) based on genetic relationship matrix (GRM).	Low	High	Yes	Can be applied to categorical and continuous environmental covariates. Needs to precompute GRM.
GxEMM [18]	Individual-level genotype	LMM that can be solved using REML, Haseman–Elston (HE) regression or phenotype-correlation-genotype-correlation.	Low- Medium	High	Yes	Flexible estimation algorithms accommodating different sample sizes. Direct liability-scale estimation for binary traits.
MTG2 [21]	Individual-level genotype	LMM, which incorporates average information algorithm and GRM eigen-decomposition techniques to speed up computation.	Medium	High	Yes	Higher computational efficiency compared to traditional REML method utilizing computational techniques.
RNM [13] (MRNM [13])	Individual-level genotype	(Multivariate) Reaction Norm Model, which distinguishes GE interaction and GE correlation.	Low	High	Yes	Distinguishes GE interaction and GE correlation. More complex model increases computational resource.
LEMMA [19] (GPLEMMA [20])	Individual level genotype	Whole genome regression method for GE interaction phenotypic variance using HE regression.	Medium	High	Yes	Incorporates multiple environmental covariates to estimate the combined GE effects. Slightly inflated type I error rate.
MonsterLM [22]	Individual level genotype	Multiple linear regression method with LD-pruned genotype data within independent genomic blocks.	Medium-High	Medium	Yes	GE polygenicity is not assumed. Potential genetic information loss in variant filtering and LD pruning.
LDSC-based methods (GxESum [7], PIGEON [8])	GWIS Summary statistics	Under the LMM model, the first moment of the squared Z-score of each variant is related to the GE variance parameter. Method of moments is used for estimation.	High	Low	No	Requires high-quality genetic reference panel. Violation of GE polygenicity leads to bias.
LDER-GE	GWIS Summary statistics	Under the LMM model, the first moment of the product Z-score of any variant pair is related to the GE variance parameter. Method of moments is used for estimation.	High	Medium	No	Higher estimation accuracy than LDSC-based methods. Requires high-quality genetic reference panel. Violation of GE polygenicity leads to bias.

Methods that employ individual-level genotype data adopt distinct estimation approaches and techniques. For instance, GCI-GREML [13] and GxEMM [19] utilize the restricted maximum likelihood (REML) method with a genetic relationship matrix to estimate GE interaction variance. In contrast, LEMMA [20] and GPLEMMA [21] estimate the proportion of GE variance using the Haseman–Elston (HE) regression. Notably, MTG2 [22] offers flexibility by adapting either REML or HE regression depending on sample size, thereby enhancing computational efficiency. MRNM [14] employs a reaction norm model to discern between GE interaction and GE correlation. While individual-level methods generally yield higher estimation accuracy, they come at the cost of increased computational burden and privacy concerns associated with the release of genotype data.

On the other hand, existing summary statistics-based methods, LDSC-based methods [7, 8, 15], offer exceptional computational efficiency, making them well suited for large-scale biobank analyses. However, they tend to provide less precise estimates compared to individual-level methods. Our proposed method, LDER-GE, represents a substantial advancement in bridging the gap between estimation accuracy while maintaining the computational efficiency and data privacy.

### Method overview

We propose LDER-GE to improve statistical efficiency with summary-level association statistics. Under the polygenic GE model where each standardized variant-by-E term has small effect, the expectation of cross-variant level GE interaction association chi-square statistics is (details in methods and supplementary note 1)


(1) \begin{equation*} E\left(Z{Z}^T\right)=N{h}_I^2L/M+\left(c+2\left({h}_I^2+{\sigma}_1^2\right)\right)R, \end{equation*}

where *Z* is the GWIS Z-score vector. Each entry is the Z score scalar for testing the marginal GxE effect separately for each variant. *R* is the LD matrix, *L* = *R^T^R* is the LD score matrix, *N* is the sample size of the GWAS summary statistics, *c* is the unconstrained intercept with potential inflation, ${\sigma}_1^2$ is the non-genetic environment interaction variance (RxE interaction proportion), and ${h}_I^2$ is the explained variance of the GE interaction. We incorporate full LD information by conducting eigen-decompose the LD matrix as *R* = *UDU^T^*, with *U* being the orthogonal matrix of eigenvectors and *D* being the diagonal eigen value matrix, and transforming the original GWIS Z-score vector *Z: Z~ = D^-1/2^U^T^Z*, yielding


(2) \begin{equation*} E\left({\overset{\sim }{Z}}_j^2\right)=N{h}_I^2{D}_{jj}/M+\left(c+2\left({h}_I^2+{\sigma}_1^2\right)\right). \end{equation*}

We run iterative weighted least squares to estimate ${h}_I^2$ and the intercept, followed by delete-block-wise jackknife to estimate the standard error.

### Simulation results using real genotype panel

**Figure 1:**
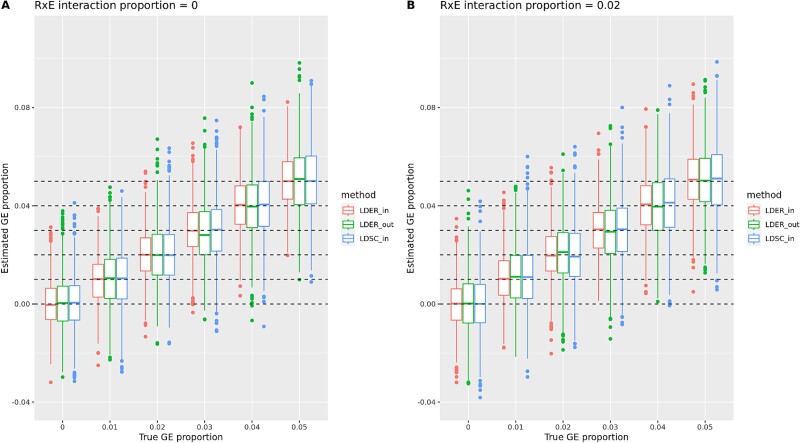
Boxplot simulation results comparison of LDER-GE and LDSC-based method with in-sample and out-sample reference panel, continuous phenotype.

Figure 1 and Table 2 compare the performance of LDER-GE and the LDSC-based method across various parameter combinations. Across all simulation scenarios, the LDER-GE method consistently had better performance than the LDSC-based method, whether utilizing a UK Biobank (UKBB) in-sample or 1000 Genomes project out-sample reference panel. “LDER-in” and “LDSC-in” mean that the genetic reference panel is constructed with the same UKBB subjects as in GWIS analysis. “LDER-out” means that the genetic panel is constructed with out-sample 4 891 000-Genome project subjects. We assessed performance using precision, which is the inverse of the standard deviation, and root mean-squared error (RMSE). The estimation efficiency of “LDER-in” is consistently higher than “LDSC-in” across all simulation scenarios, with precision increasing from 19% to 24%, and the average increase is 23%. Notably, this precision improvement was, on average, equivalent to a 51% increase (explained in Supplementary Note 2) in sample size when analyzing continuous simulated phenotypes during in-sample estimation. LDER-GE using out-sample 1000 Genome project reference panel also exhibits higher statistical efficiency than LDSC-based methods, although at smaller improvement than LDER-GE with in-sample UKBB reference panel (Table 2). Table 2 shows that the type-I error rates were well controlled for all three methods: LDER-GE using an in-sample LD panel, LDER-GE using an out-sample LD panel, and LDSC-based in-sample LD panel. This held true both in scenarios with and without the presence of nongenetic residual–environment interaction effects. While controlling the type-I error rate at the same level, Table 2 presents the statistical power to test the GE interaction effect at different effect sizes, and LDER-GE is systemically more powerful than LDSC-based methods, because of higher statistical efficiency. Our model does not assume that the exposure is normally distributed [equation (4), Supplementary note 1]. To validate this, we conducted simulations using balanced and unbalanced binary exposure with prevalence at 0.5 and 0.05 separately. LDER-GE, remaining the same as other simulation scenarios, has systematically higher estimation accuracy, statistical power, and a well-controlled type-I error rate (Table S1, Fig. S1Supplementary Note 2).

**Table 2:** Simulation results of LDER-GE and LDSC-based methods for continuous phenotypic GE interaction variance proportion. The values on the left side of separation symbol “|” are with RxE proportion = 0, and the values on the right side of separation symbol “|” are with RxE proportion = 0.02. Each simulation scenario has 1000 replications

H_I_^2a^	Method	Average	Precision^b^	RMSE^c^	Positive^d^
***0***	***LDER_in***	***0.000 | -0.000***	***106.756 | 102.616***	***0.0094 | 0.0097***	***0.049 | 0.045***
0	LDER_out	0.000 | 0.000	92.903 | 89.515	0.0108 | 0.0112	0.047 | 0.050
0	LDSC_in	0.001 | 0.000	89.673 | 84.343	0.0112 | 0.0119	0.048 | 0.049
***0.01***	***LDER_in***	***0.010 | 0.010***	***100.261 | 95.100***	***0.0100 | 0.0105***	***0.148 | 0.141***
0.01	LDER_out	0.010 | 0.011	86.622 | 81.768	0.0116 | 0.0123	0.136 | 0.133
0.01	LDSC_in	0.010 | 0.010	80.488 | 77.226	0.0124 | 0.0130	0.126 | 0.124
***0.02***	***LDER_in***	***0.020 | 0.020***	***97.384 | 93.858***	***0.0103 | 0.0107***	***0.470 | 0.427***
0.02	LDER_out	0.020 | 0.021	83.097 | 81.217	0.0120 | 0.0123	0.355 | 0.357
0.02	LDSC_in	0.020 | 0.020	78.530 | 76.540	0.0127 | 0.0131	0.308 | 0.296
***0.03***	***LDER_in***	***0.030 | 0.030***	***92.364 | 90.224***	***0.0108 | 0.0111***	***0.810 | 0.759***
0.03	LDER_out	0.029 | 0.029	79.431 | 76.348	0.0126 | 0.0131	0.636 | 0.612
0.03	LDSC_in	0.030 | 0.030	75.790 | 73.313	0.0132 | 0.0136	0.618 | 0.589
***0.04***	***LDER_in***	***0.040 | 0.040***	***87.793 | 84.222***	***0.0114 | 0.0119***	***0.948 | 0.935***
0.04	LDER_out	0.039 | 0.040	75.311 | 72.691	0.0133 | 0.0138	0.860 | 0.818
0.04	LDSC_in	0.041 | 0.041	72.198 | 68.858	0.0139 | 0.0146	0.844 | 0.818
***0.05***	***LDER_in***	***0.050 | 0.051***	***87.591 | 83.830***	***0.0114 | 0.0119***	***0.995 | 0.990***
0.05	LDER_out	0.051 | 0.051	73.318 | 73.818	0.0136 | 0.0136	0.959 | 0.951
0.05	LDSC_in	0.051 | 0.051	69.015 | 67.876	0.0145 | 0.0147	0.947 | 0.935

a True GE interaction variance proportion.

b Inverse of empirical standard deviation.

c RMSE rate.

d Positive test rate over 1000 replications. When RxE proportion = 0, it represents false positive rate. When RxE proportion > 0 (right side of separation symbol “|”), it represents statistical power. For 1000 simulations, the 1-unit standard error of type-I error rate estimate at 0.05 is sqrt(0.05^*^0.95/1000) = 0.0069.

*Highlighted method:* highest precision, lowest RMSE and highest statistical power (only applicable when H_I_^2^ > 0) among the three methods.

We also included SNPs with MAFs (between 0.01 and 0.05) in the simulations to examine the sensitivity of LDER-GE with complex and noisy LD patterns. All simulation settings and scenarios were the same as those for SNPs with in MAF > 0.05. The results and details can be found in Supplementary note 2. To summarize, the in-sample estimation performance of LDER-GE is consistently better than LDSC-based methods across different simulation scenarios, as we observed for variants with MAF > 0.05. However, for the same simulation parameter setting, the in-sample estimation precision with SNPs has low MAFs is lower than that for SNPs with MAF > 0.05 for both LDER-GE and LDSC-based methods. While the out-sample performance of LDER-GE degrades with the inclusion of SNPs with low MAFs in terms of bias and accuracy, due to unreliable LD estimates from the small sample size of the 1000 Genomes project [23]. We recommend use MAF > 0.05 variants and an in-sample or a large sample size reference panel where applicable to ensure the best overall estimation performance.

The transformation from observed-scale variance to liability-scale variance, as achieved by the Robertson transformation [24], relies on the normality assumption of the liability distribution. However, this assumption is violated with the non-normal GE term. Our simulations, considering various prevalence and GE interaction variance settings, suggest that the transformation provides reasonably unbiased results (Fig. S2B–DSupplementary Note 2) when the disease is not rare, with a prevalence exceeding 10%, or when the GE interaction variance remains relatively small, at or below 5%. Nevertheless, in cases where the disease is rare, and the GE interaction variance is nontrivial (Fig. S2A and BSupplementary Note 2), the transformed liability-scale GE interaction variance estimate tends to be overestimated. This trend becomes more pronounced as the disease prevalence decreases. Despite the potential bias introduced by the Robertson transformation [24] in presence of the GE term, the type-I error rate for tests involving binary phenotypes remained well controlled (Table 3) across varying prevalence settings. Our findings related to the estimation of GE liability-scale variance and the results of type-I error rate simulations for binary phenotypes align with a previous study [8], except that LDER-GE achieved better estimation accuracy than LDSC-based method using in-sample and out-sample LD reference panels (Table S2Supplementary Note 2). Note that in a case–control study where the sample prevalence is different from the population prevalence, Sang Hong Lee’s transformation [25], which additionally adjusts for the oversampling of cases, should be used.

**Table 3:** Type-I error rate at 0.05 level for LDER-GE and LDSC-based method for binary phenotype simulation scenarios, each scenario with 1000 replications. For 1000 simulations, the 1-unit standard error of type-I error rate estimate at 0.05 is sqrt(0.05^*^0.95/1000) = 0.0069. In each cell, the values on the left side of separation symbol “|” are obtained using linear regression, and the values on the right side of separation symbol “|” are obtained using logistic regression

Method	Prevalence	Type-I error rate
LDER_in	0.05	0.052 | 0.053
	0.1	0.043 | 0.047
	0.2	0.052 | 0.050
	0.3	0.047 | 0.048
	**Average**	**0.049 | 0.050**
LDER_out	0.05	0.055 | 0.052
	0.1	0.046 | 0.050
	0.2	0.051 | 0.049
	0.3	0.047 | 0.050
	**Average**	**0.050 | 0.050**
LDSC_in	0.05	0.050 | 0.051
	0.1	0.045 | 0.048
	0.2	0.055 | 0.055
	0.3	0.048 | 0.050
	**Average**	**0.049 | 0.051**

Average: the averaged value across different prevalences.

The estimation formula is derived assuming the Z-scores are obtained from a linear regression model even for binary phenotypes. However, most binary phenotype summary statistics are reported on the log-odds scale, which leads to inaccurate results [26]. When sample size is large, logistic regression summary statistics yields similar results as linear regression [26]. We conducted sensitivity analysis to validate this conclusion in light of GxE analysis. We obtained summary statistics using logistic regression and results were almost the same as linear regression in terms of estimation accuracy, RMSE, false positive rate, power, and boxplot shape (Fig. S3Supplementary Note 2, Table S2).

### Real data analysis using UK Biobank data

**Figure 2:**
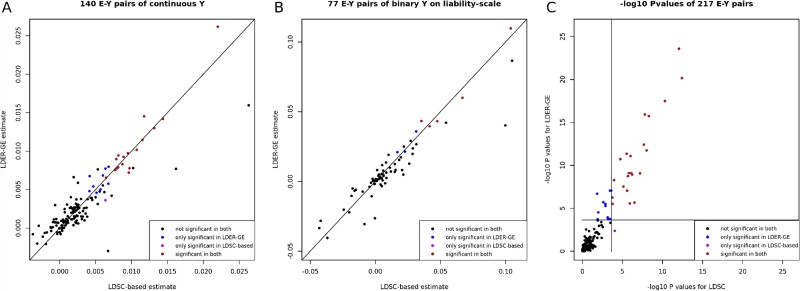
GE interaction analysis results from LDSC-based method and LDER-GE of 217 environmental covariate phenotype pairs in UKBB dataset. (A) Estimated ${h}_I^2$ values for continuous phenotypes. B: Estimated ${h}_I^2$ values for binary phenotypes reported on the liability-scale. (C) Log *P* values of LDER-GE and LDSC-based method. Two lines are significance cutoff at 0.05/217.

We examined 217 E-Y pairs involving 307 259 unrelated European ancestry individuals and a total of 966,766 genetic variants from the UKBB. We employed both the LDER-GE and LDSC-based methods. Following Bonferroni correction, LDER-GE identified 34 significant pairs, of which 22 overlapped with the 23 pairs identified by the LDSC-based method (Fig. 2A and B). Further details about the 12 E-Y pairs exclusively identified by LDER-GE and the one E-Y pair uniquely identified by the LDSC-based method can be found in Table S3Supplementary Note 2. We conducted literature search for the 12 E-Y pairs identified only by LDER-GE, and we summarized GE interaction evidence on 10 pairs (Table 4). Two polygenic-risk-score-based (PRS) studies [27, 28] evidenced gene–age interaction on coronary artery disease (CAD) and one genetic-variant-level study [29] proved on triglycerides (TGs). Gene–age interaction on blood pressure was discovered through extensive GWAS data from three blood pressure consortia [30] and linkage analysis [31]. In our analysis, LDER-GE successfully captured signals from both systolic blood pressure (SBP) and diastolic blood pressure (DBP), while the LDSC-based method failed to detect the DBP signal. Gene–alcohol–drinking interaction was shown on blood glucose level and type-II diabetes (T2D) via one PRS-based study [32] and two genetic-variant-level studies [33, 34]. Additionally, gene–sex interaction effects have been reported for traits such as APOB [35, 36], height [37], total cholesterol level [35, 38, 39], height [40, 41], psychiatric health [42, 43], and T2D [44, 45], all of which were exclusively detected by LDER-GE.

**Table 4:** Literature search evidence of the 12 E-Y pairs identified only by LDER-GE

Environmental covariate	Phenotype	Literature DOI	Study methods
AGE	CAD	https://doi.org/10.1161/circ.141.suppl_1.P457 https://doi.org/10.1371/journal.pgen.1009723	PRS-based PRS-based
AGE	SBP	https://doi.org/10.1016/j.ajhg.2014.05.010 https://doi.org/10.1161/HYPERTENSIONAHA.108.120071	GWAS/genetic variant level Linkage analysis/eQTL/genetic variant level
AGE	TG_norm	https://doi.org/10.1186/1471-2156-14-33	GWAS/genetic variant level
Alcohol_intake_frequency	HbA1c	https://doi.org/10.1186/s12986-019-0396-x https://doi.org/10.1038/s41598-019-56011-y	PRS-based Survival analysis/genetic variant level
Alcohol_intake_frequency	T2D	https://doi.org/10.1186/s12986-019-0396-x https://doi.org/10.1038/s41598-019-56011-y https://doi.org/10.1139/apnm-2017-023	PRS-based Survival analysis/genetic variant GWAS/genetic variant level
SEX	ApolipoproteinB	https://doi.org/10.1186/1476-511X-11-61 https://doi.org/10.1194/jlr.P035238	GWAS/genetic variant level GWAS/genetic variant level
SEX	CHO_norm	https://doi.org/10.1186/s12944-022-01736-5 https://doi.org/10.1186/1476-511X-11-61 https://doi.org/10.3390/nu15204385	GWAS/genetic variant level GWAS/genetic variant level PRS-based
SEX	height	https://doi.org/10.1016/j.xgen.2023.100297 https://doi.org/10.1038/srep28496	Amplification model Twin analysis
SEX	Neuro_score	https://doi.org/10.1016/j.biopsych.2020.12.024 https://doi.org/10.1016/j.semcdb.2017.10.016	GWAS/genetic variant level Review
SEX	T2D	https://doi.org/10.1186/s13293-023-00521-y https://doi.org/10.1007/s00125-006-0375-4	GWAS/genetic variant level Linkage analysis
SEX	health_rating	NA	NA
townsendscore	health_rating	NA	NA

NA: no replicated articles found, due to limited direct studies on “health_rating”.

**Figure 3:**
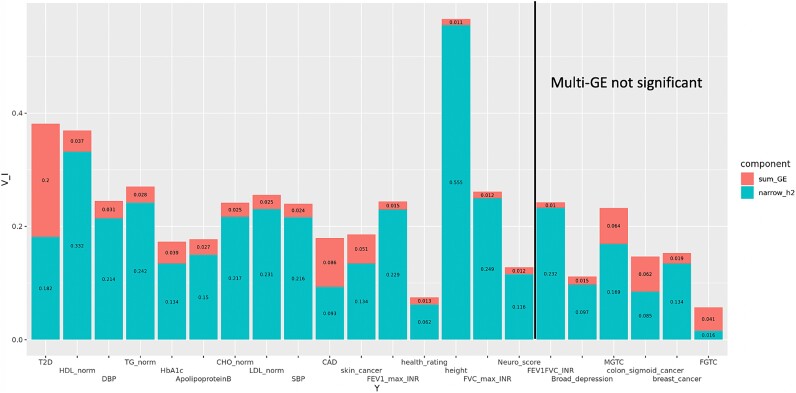
Phenotypic variance explained by narrow-sense heritability and aggregated multi-GE interactions for the 22 phenotypes, ordered by increasing *P* value of multi-GE interactions. Right of the solid black line, six phenotypes are not significant. For binary phenotypes, proportion is reported on the liability scale. FGTC: female genital tract cancer. MGTC: male genital tract cancer.

In summary, the estimated values obtained using LDER-GE and the LDSC-based method exhibited strong overall consistency. However, we note that the standard error of LDER-GE estimates was, on average, 21% lower than that of the LDSC-based method, a result consistent with our simulation findings. The narrower confidence interval range leads to systematically lower *P* values and higher statistical power (Fig. 3). A comprehensive overview of the analysis results for all 217 E-Y pairs can be found in Table S4Supplementary Note 2. Among the 10 environmental covariates investigated, sex, body mass index (BMI), and age exhibited relatively larger genome-level GE interaction effects and lower *P* values compared to the other covariates (Table S4Supplementary Note 2). On the other hand, the pollution covariate pm2.5 did not exhibit statistically significant GE interactions across all tested phenotypes, including several lung-related traits. This can be a consequence of small GE interaction magnitudes and inaccuracy of air pollution indicator measurements.

For each of the 22 phenotypes, we estimated the aggregated multi-GE interaction variance using ordered (Methods) covariates: age, sex, BMI, packed years of smoking, Townsend deprivation index, alcohol intake frequency. Post-Bonferroni correction, the LDSC-based method identified 13 phenotypes, while LDER-GE identified 16 phenotypes that covered all the 13 phenotypes discovered by LDSC-based method (Table S5, Fig. S4Supplementary Note 2). The additional three phenotypes identified are height, normalized Forced Vital Capacity (FVC) and neuroticism score, whose aggregated GE interaction variances were estimated around 1%. Notably, some phenotypes like depression and male genital tract cancer exhibited significant single-covariate GE interactions but were not detected with multi-GE interaction variance. This discrepancy may be due to noise introduced by other weak covariates during multicovariate aggregation. Similar to single-covariate GE analysis, LDER-GE consistently yielded results comparable to the LDSC-based method in assessing multi-GE interaction variance but reported an average standard error 21% smaller than the LDSC-based method. We also estimated narrow-sense heritability *h*^2^ and compared it to the aggregated multi-GE interaction variance. Notably, 13 of the 22 phenotypes exhibited substantial multi-GE interaction variance, contributing >10% relatively to the narrow-sense heritability (Table 5). For conditions like type-II diabetes (T2D) and coronary artery disease (CAD), the multi-GE interaction variance approached the magnitude of the narrow-sense heritability, providing valuable insights into disease etiology. However, we caution that when interpreting results for binary diseases, as the liability-scale transformation may introduce bias. We reversed the covariate order and used the set (alcohol intake frequency, Townsend deprivation index, packed years of smoking, BMI, sex, age) for the same analysis. It turned out that the order of covariates did not substantially affect the results (Table S6).

**Table 5:** Aggregated multi-GE interaction variance and narrow sense heritability (h^2^) comparison of the 22 phenotypes

Y	GE_variance	SE	*P*	*h* ^2^	GE_variance/h^2^
T2D	2.00E-01	1.95E-02	1.33E-24	1.82E-01	1.10E+00
HDL_norm	3.72E-02	3.81E-03	1.60E-22	3.32E-01	1.12E-01
DBP	3.11E-02	3.32E-03	7.32E-21	2.14E-01	1.45E-01
TG_norm	2.84E-02	3.43E-03	1.34E-16	2.42E-01	1.17E-01
HbA1c	3.90E-02	4.78E-03	3.74E-16	1.34E-01	2.91E-01
ApolipoproteinB	2.75E-02	3.40E-03	5.83E-16	1.50E-01	1.83E-01
CHO_norm	2.50E-02	3.34E-03	6.93E-14	2.17E-01	1.15E-01
LDL_norm	2.52E-02	3.38E-03	9.70E-14	2.31E-01	1.09E-01
SBP	2.38E-02	3.26E-03	2.70E-13	2.16E-01	1.10E-01
CAD	8.59E-02	1.89E-02	5.19E-06	9.33E-02	9.21E-01
skin_cancer	5.11E-02	1.18E-02	1.49E-05	1.34E-01	3.80E-01
FEV1_max_INR	1.46E-02	3.69E-03	7.44E-05	2.29E-01	6.36E-02
health_rating	1.29E-02	3.28E-03	8.94E-05	6.21E-02	2.07E-01
height	1.10E-02	3.09E-03	3.80E-04	5.55E-01	1.98E-02
FVC_max_INR	1.21E-02	3.47E-03	5.09E-04	2.49E-01	4.84E-02
Neuro_score	1.18E-02	3.66E-03	1.29E-03	1.16E-01	1.02E-01
FEV1FVC_INR	1.00E-02	3.40E-03	3.14E-03	2.32E-01	NA
Broad_depression	1.45E-02	4.96E-03	3.45E-03	9.69E-02	NA
male_genital_tract_cancer	6.37E-02	2.30E-02	5.63E-03	1.69E-01	NA
colon_sigmoid_cancer	6.22E-02	3.49E-02	7.45E-02	8.49E-02	NA
breast_cancer	1.89E-02	1.59E-02	2.35E-01	1.34E-01	NA
female_genital_tract_cancer	4.11E-02	5.25E-02	4.34E-01	1.61E-02	NA

NA: the tested GE_variance is non-significant.

### Computational efficiency

The whole computation process was divided into LD preparation part and estimation part [16]. LD preparation was based on 10 000 UKBB subjects and 396 330 variants. We used processor Intel(R) Xeon(R) Gold 6326 CPU @ 2.90GHz with 32 CPUs for computation tasks. Each computation was repeated 50 times. LDSC does not support for parallel computing, so we split the genome into chromosomes and run the LD calculation independently. Overall, LDER-GE is slightly slower than LDSC in LD preparation and estimation, but the difference is not very substantial, as the estimation time elapsed is at minutes level (Table S8). Both LDER-GE and LDSC can conduct different GE analysis without recalculating the LD information.

## Discussion

In this study, we introduce LDER-GE to improve the precision of estimating GE interaction variance of complex traits using summary statistics. LDER-GE leverages full LD information from the LD panel, while LDSC-based methods [7, 8] rely solely on the squared LD panel's diagonal information. Our simulations and analysis of UKBB data demonstrate LDER-GE's superiority over LDSC-based approaches in terms of estimation accuracy and RMSE. LDER-GE's improved accuracy enables the detection of more genome-level GE interactions that might go undetected by LDSC-based methods.

From real data analysis, sex and BMI had more detectable GE interaction effects over various health-related traits, consistent with results from other studies. For example, several studies reported that sex modifies genetic effects on lipid traits [29], obesity [46, 47], and hypertension [48] from different perspectives. The BMI is known to be causal to multiple health-related traits such as T2D and hypertension [49, 50], part of which could be reasoned from the GE interaction effects [8]. While biological sex is almost fixed for most individuals in the population throughout the lifetime as well as its associated GE interaction variance, the BMI varies during different life stages. The gene–BMI interaction study potentially brings additional significance to the clinical prevention or treatment to diseases such as T2D and hypertension, given their considerable GE interaction variance estimate. The higher statistical efficiency of LDER-GE allows us to better estimate the aggregated multi-GE interaction variance, which is comparable to narrow-sense heritability, especially for T2D and coronary artery disease (CAD). However, such interpretation must be accompanied with caution, due to the normality and additive effect assumption violations of Robertson transformation [24, 25, 51]. Empirically, simulations demonstrate the true aggregated multi-GE interaction variance is lower than the transformed liability-scale variance when the disease is rare (≤5%), but the magnitude difference is not considerable.

Our real data analysis highlighted the significant gene by SEX and gene by BMI interaction effects over various health-related traits, a finding consistent with diverse studies. For instance, sex has been shown to modify genetic effects on traits like lipid [29], obesity [46, 47], and hypertension [48]. Additionally, BMI, a known causal factor for multiple health-related traits such as T2D and hypertension [49, 50], may exert some of its influence through gene–environment (GE) interactions [8]. We emphasize the importance identifying the correct causal direction from the environmental covariate to the phenotype [52], for example, BMI to various health outcomes. While biological sex remains relatively constant throughout an individual's lifetime, BMI is subject to regulation. Investigating BMI's GE interaction implications could have clinical significance, particularly in preventing or treating diseases like T2D and hypertension, given their substantial GE interaction variance estimates. LDER-GE's enhanced statistical efficiency enables more accurate estimation of aggregated multi-GE interaction variance, which, notably, often approaches the magnitude of narrow-sense heritability, especially for T2D and CAD. However, it's crucial to exercise caution in interpreting these findings, as they hinge on the normality and additive effect assumptions of the Robertson transformation [24, 25, 51]. Empirical simulations reveal that in cases of rare diseases (prevalence ≤5%), the true aggregated multi-GE interaction variance tends to be lower than the estimated liability-scale variance, though the difference in magnitude is not substantial.

As previously discussed [8], it's not recommended to directly estimate the nongenetic-residual–environment interaction variance from the intercept using the formula (intercept – 2^*^h_I_ [2])/2. This is because the intercept can be inflated by factors such as population stratification and other confounding effects, making it difficult to separate from the nongenetic-residual-environment interaction variance. When analyzing binary phenotypes, there's the additional challenge of unknown prevalence differences between the sampling population and the target population, which can further inflate the estimated intercept [15].

To expedite computation, we partitioned the entire genome into 1009 approximately independent blocks based on their LD relationships. We derived the block-wise LD matrix using a dataset of 307 259 individuals of European ancestry from the UKBB. Alternatively, we also calculated the out-sample LD matrix using 489 subjects from the 1000 Genomes project [53], employing a linear shrinkage method [54, 55], and the 1703 genomic blocks from LDetect [56]. Typically, the 1000 Genomes project reference panel is utilized when in-sample LD information is unavailable. For readers' convenience, both computed panels are accessible online. Our simulation results underscore the robustness of LDER-GE, whether LD panels are constructed from the UKBB or the 1000 Genomes project. However, it's advisable to prioritize the UKBB reference when there's a significant overlap between the variants in the GWIS input summary statistics and the UKBB reference panel, mainly due to its larger sample size.

As an extension of the LDSC-based method, LDER-GE inherits most of its limitations. Firstly, it assumes polygenic GE effects on the phenotype, and a violation of this assumption can result in underestimation of the variance component [57]. Secondly, LDER-GE needs a reasonably large reference panel to estimate LD, especially for SNPs with low MAFs. Thirdly, our model does not differentiate between GE covariate correlations, potentially introducing estimation bias due to overadjustment [8]. Methods addressing such correlations are available [14]. While efforts are being made to address these limitations, future research could explore incorporating variant functional annotation or allele frequency information to enhance estimation [57]. We note a recently proposed Mendelian randomization-like approach [12] integrating GWAS main effects to uncover SNP-level GxE effects through structural equation modeling. It was shown that the SNP-level results can be used to estimate genome-level GxE proportions by incorporating LD. The eigen decomposition technique of LDER-GE may be used to aggregate full LD information to further improve the power of this method.

To summarize, LDER-GE utilizes full LD information and summary statistics to estimate the phenotypic variance explained by GE interactions more accurately than LDSC-based estimation methods. LDER-GE controls the computational burden and time well compared to methods that requires individual-level data input and can be employed to estimate multiple E-Y pairs of large sample size.

## Materials and methods

### LDER-GE modeling & estimation

We consider the following model


(3) \begin{equation*} {\boldsymbol{Y}}_{\boldsymbol{i}}={\sum}_{\boldsymbol{j}=\mathbf{1}}^{\boldsymbol{M}}{\boldsymbol{G}}_{\boldsymbol{j}\boldsymbol{i}}{\boldsymbol{\beta}}_{\boldsymbol{j}}+{\sum}_{\boldsymbol{j}=\mathbf{1}}^{\boldsymbol{M}}{\boldsymbol{S}}_{\boldsymbol{j}\boldsymbol{i}}{\boldsymbol{\gamma}}_{\boldsymbol{j}}+{\boldsymbol{\epsilon}}_{\mathbf{1}\boldsymbol{i}}{\boldsymbol{E}}_{\boldsymbol{i}}+{\boldsymbol{\epsilon}}_{\mathbf{0}\boldsymbol{i}}, \end{equation*}

where *Y_i_* is the phenotype residual for subject *i* already adjusted for fixed effects including the exposure covariate effects. *E_i_* is the standardized exposure covariate for subject *i*. Suppose there are *M* variants. *G_ji_* is the standardized *j*th variant for subject *i*. S_ji_ = G_ji_*E_i_ is the GE interaction product term for variant *j* of subject *i*, *ϵ_1i_* is the nongenetic residual that has exposure interaction effect. ϵ_0i_ is the residual independent from all other parts, *β_j_* is the true additive genetic effect for variant *j*, *γ_j_* is the true interaction effect for variant *j*. Following PEGION’s^7^ setting, we model *β_j_* and *γ_j_* using the following random effects model:


$$ {\boldsymbol{\beta}}_{\boldsymbol{j}}\sim \boldsymbol{N}\left(\mathbf{0},{\boldsymbol{h}}_{\boldsymbol{g}}^{\mathbf{2}}/\boldsymbol{M}\right), $$

$$ {\boldsymbol{\gamma}}_{\boldsymbol{j}}\sim \boldsymbol{N}\left(\mathbf{0},{\boldsymbol{h}}_{\boldsymbol{I}}^{\mathbf{2}}/\boldsymbol{M}\right), $$

where ${\boldsymbol{h}}_{\boldsymbol{g}}^{\mathbf{2}}$ is the narrow-sense heritability and ${\boldsymbol{h}}_{\boldsymbol{I}}^{\mathbf{2}}$ is the GE interaction variance that we are interested in estimating. *β_j_* and *γ_j_* may or not be correlated. We model *ϵ_0_* and *ϵ_1_* using random effects model:


$$ {\boldsymbol{\epsilon}}_{\mathbf{0}}\sim \boldsymbol{N}\ \left(\mathbf{0},{\boldsymbol{\sigma}}_{\mathbf{0}}^{\mathbf{2}}\right), $$

$$ {\boldsymbol{\epsilon}}_{\mathbf{1}}\sim \boldsymbol{N}\ \left(\mathbf{0},{\boldsymbol{\sigma}}_{\mathbf{1}}^{\mathbf{2}}\right), $$

where ${\boldsymbol{\sigma}}_{\mathbf{1}}^{\mathbf{2}}$ is the nongenetic environment interaction variance (RxE interaction proportion). Again, *ϵ_0_* and *ϵ_1_* may or may not be correlated with the data-generating process set up in equation (3).

In a marginal linear regression model for variant *j* with GE interaction effect:


(4) \begin{equation*} {\boldsymbol{Y}}_{\boldsymbol{res}}\sim{\boldsymbol{\mathrm{\beta}}}_{\boldsymbol{gj}}{\boldsymbol{G}}_{\boldsymbol{j}}+{\boldsymbol{\beta}}_{\boldsymbol{Ij}}{\boldsymbol{S}}_{\boldsymbol{j}}+\boldsymbol{\mathrm{\varepsilon}}, \end{equation*}

For variant *j*, assuming variants and the environmental covariate are standardized, its Z-score to test GE interaction effect $ {\boldsymbol{\beta}}_{\boldsymbol{Ij}} $ is:


(5) \begin{equation*} {\boldsymbol{Z}}_{\boldsymbol{j}}={\sum}_{\boldsymbol{i}=\mathbf{1}}^{\boldsymbol{N}}{\boldsymbol{S}}_{\boldsymbol{j}\boldsymbol{i}}{\boldsymbol{Y}}_{\boldsymbol{i}}/\sqrt{\boldsymbol{N}}={\sum}_{\boldsymbol{i}=\mathbf{1}}^{\boldsymbol{N}}{\boldsymbol{G}}_{\boldsymbol{j}\boldsymbol{i}}{\boldsymbol{E}}_{\boldsymbol{i}}{\boldsymbol{Y}}_{\boldsymbol{i}}/\sqrt{\boldsymbol{N}}, \end{equation*}

Under the polygenic GE model, we derive (supplementary note 1), in matrix form, that


(6) \begin{equation*} E\left(Z{Z}^T\right)=N{h}_I^2L/M+\left(c+\left(K(E)-1\right)\left({h}_I^2+{\sigma}_1^2\right)\right)R, \end{equation*}

(7) \begin{equation*} E\left(Z{Z}^T\right)=N{h}_I^2L/M+\left(c+2\left({h}_I^2+{\sigma}_1^2\right)\right)R,\qquad\qquad \end{equation*}

where *Z* is the GWIS Z-score vector with each entry being the Z score scalar for testing the marginal GxE effect in a linear regression model separately for each variant. *R* is the LD matrix, *L = R^T^R* is the LD score matrix, *N* is the sample size of the GWAS summary statistics, *c* is the unconstrained intercept with potential inflation, and *K(E)* is the kurtosis of the exposure covariate.

In the case of *E* being standard normal, equation (6) reduces to the equation (7). Following the original LDER [16] framework, we eigen-decompose the LD matrix as *R = UDU^T^*, where *U* is the orthogonal matrix of eigenvectors and *D* is the diagonal eigen value matrix. Then, we transform the original GWIS Z-score vector Z*: Z~ = D^-1/2^U^T^Z* and have


(8) \begin{equation*} E\left({\overset{\sim }{Z}}_j^2\right)=N{h}_I^2{D}_{jj}/M+\left(c+2\left({h}_I^2+{\sigma}_1^2\right)\right). \end{equation*}

The transformed summary statistic vector contains all LD information, and the estimation efficiency is improved compared to LDSC-based methods consequently. The estimation task is accomplished using the iterative least squares and standard error is estimated using delete-block-wise jackknife. We then calculate the *P* value and conduct statistical inference using the estimated GE proportion and the estimated standard error.

To analyze binary outcomes, we transformed the observed-scale heritability to liability-scale heritability using Robertson transformation [24]. It has been pointed out that when the GE interaction variance is large, the normality assumption of the phenotype liability may be violated, resulting in biased results of Robertson transformation [8, 58]. However, our simulation results showed that when the GE interaction variance proportion was small, Robertson transformation still yielded a reasonably accurate result (Fig. S2, Table S2), consistent with previous studies [8].

The regression weight of the transformed summary statistics vector takes the same form as LDER [16] except the additional intercept inflation component (Supplementary note),


(9) \begin{equation*} {w}_i=\mathit{\min}\left({D}_{ii},1\right)/{\left(N{h}_I^2{D}_{ii}/M+1+2\left({h}_I^2+{\sigma}_1^2\right)\right)}^2, \end{equation*}

where ${\left(1+2\left({h}_I^2+{\sigma}_1^2\right)+N{h}_I^2{D}_{ii}/M\right)}^2$ is proportional to the variance of *Z~_i_* (Supplementary Note 2) and the shrinkage operation $\mathit{\min}\left({D}_{ii},1\right)$ reduces the noise from big eigenvalues from LD matrix with lower sample sizes.

### UKBB data for simulation and real data analysis

The research conducted in this study utilized data from the UKBB Resource. The genomic partitioning and simulation analysis was conducted using UKBB dataset with application number 29900. The real data analysis was conducted using UKBB dataset with application number 32285. Detailed information regarding data access, ethical approval, quality control procedures, and phenotype definitions can be found in the supplementary note 2.

### Reference panel construction

We first took the intersection between UKBB^18^ imputed genotype panel, 1000 Genomes project [53] genotype panel and hapmap3 project [59] variant list, resulting in *M* = 396 330 common variants. Then, we partitioned the entire human genome into 1009 roughly independent blocks using the panel of 396 330 common variants from UKBB European ancestry (*N* = 276 050). We partitioned the genome such that the linked SNP pairs (absolute LD coefficient |r| > 1.96/sqrt(276050) = 0.00373) are within 100 kilobases within each block. For simulations, in-sample reference panel was constructed using the intersected UKBB genotype panel (*N* = 276 050, *M* = 396 330) and the 1009 genomic blocks. The out-sample reference panel was constructed using the same set of variants but from the 1000 Genome project genotype panel (*N* = 489, *M* = 396 330), with the genome partition being the 1703 genomic blocks generated previously [16] for reducing the noise of low sample size. A linear shrinkage method [54, 55] was employed for out-sample reference panel construction to further reduce the noise. For UKBB real data analysis, the in-sample reference panel was constructed using the union of UKBB imputed genotype panel and UKBB array genotype panel, intersected with hapmap3 project [59] variant list (*N* = 307 259, *M* = 966 766) and the 1009 genomic blocks. For real data analysis, the variant inclusion criteria were (i) imputation score > 0.8; (ii) minor allele frequency > 0.05; (iii) missing rate < 0.01; (iv) Hardy–Weinberg Equilibrium *P*-value >5^*^10^−8^. The details of quality control procedure of simulation dataset and real analysis dataset can be found in the supplementary materials. The LDER-GE framework uses R to conduct the LD matrix eigen decomposition. We exclude the LD principal component with eigen values smaller than 10^−6^ to avoid the noise from trivial components.

### Simulations

The data generation process followed equation (10), with narrow-sense heritability h^2^_g_ fixed at 0.2, GE interaction contribution proportion h^2^_I_ varying from 0 to 0.05, non-genetic-residual–covariate interaction variance σ_1_ [2] being 0 or 0.02, E is standard normal:


(10) \begin{equation*} {Y}_i={\sum}_{j=1}^M{G}_{ji}{\beta}_j+{\sum}_{j=1}^M{S}_{ji}{\gamma}_j+{\epsilon}_{1i}{E}_i+{\epsilon}_{0i}, \end{equation*}

For each simulation from the pool of intersected UKBB genotype panel (*N* = 276 050, *M* = 396 330), we randomly chose 30 000 subjects and 19 816 (5%) causal GE variants for data generation, association analysis (linear regression on *M* = 396 330 variants using PLINK2 [60]) and GE interaction variance estimation analysis. Each parameter combination had 1000 replicated simulations. To simulate the binary outcome, we used the liability model based on corresponding critical cutoff with respect to the specified population prevalence.

### Single-covariate gene–environment interaction variance analysis of UK Biobank

We ran GWIS analysis through the “--glm interaction --variance-standardize” command of PLINK2 [60] pre-adjusted for age, sex, 40 genetic PCs, and the specific environmental covariate of interest if not age or sex using linear regression. We analyzed 22 phenotypes and 10 environmental covariates, resulting in a total of 217 (22^*^10–3) E-Y pairs with the 3 sex-specific phenotypes. The 22 phenotypes included 14 continuous phenotypes and 8 binary phenotypes. LDER-GE and LDSC-based analysis were conducted using the resulted GWIS summary statistics and pre-computed LD information.

### Aggregated multicovariate GE interaction variance analysis of UK Biobank

Suppose there is a covariate set of interest (A, B, C, …), we first run linear regression of B ~ A to get residuals of B net A: B|A, being independent from A, and we run another linear regression of C ~ A + B to get residuals of C net A and B: C|A & B, being independent from A and B. We continue the process until all residuals are independent from each other. We then run single-covariate GE interaction variance analysis on each residuals the same way but preadjust for age, sex, 40 genetic PCs and all covariates in the set excluding age and sex. By eliminating the dependency of covariates, the estimated single-covariate GE interaction variances are independent, and we summed up the estimated GE interaction variances and their variances of estimation to conduct straightforward statistical test. We explored the set (age, sex, BMI, packed years of smoking, Townsend deprivation index, alcohol intake frequency) to capture more missing heritability explained by GE interactions because these six covariates yielded nonminimally significant GE interaction signals at *P* < .05 on more than three phenotype (Table S7). The narrow-sense heritability of each phenotype was estimated using LDER and the main genetic effect GWAS summary statistics of the same UKBB cohort as GE analysis.

**Key Points**

- We reviewed existing statistical tools to estimate phenotypic variance explained by GE interactions
- We proposed a novel statistical method LDER-GE to more accurately estimate phenotypic variance explained by GE interactions while managing computational burden
- We showed that the statistical efficiency of LDER-GE is 23% higher on average than competing methods through extensive simulations
- We applied LDER-GE to real data analysis of UK-Biobank dataset and studied 22 phenotypes and 10 environmental covariates. LDER-GE detected 48% more genome-level GE interaction signals than competing methods.

## Supplementary Material

LDERGEsup2_final_bbae335

GEinteraction_supplementary_tables_round1_bbae335

LDERGEsup1_round2_bbae335

## Data Availability

“LDER-GE” and pre-computed LD information can be found at https://github.com/dongzhblake/LDER-GE. The UK-Biobank dataset can be applied at https://www.ukbiobank.ac.uk/enable-your-research/apply-for-access
